# Automated multiplex nucleic acid tests for rapid detection of SARS-CoV-2, influenza A and B infection with direct reverse-transcription quantitative PCR (dirRT-qPCR) assay in a centrifugal microfluidic platform

**DOI:** 10.1039/d0ra04507a

**Published:** 2020-09-15

**Authors:** Minghui Ji, Yun Xia, Jacky Loo, Lang Li, Ho-Pui Ho, Jianan He, Dayong Gu

**Affiliations:** a School of Nursing, Nanjing Medical University Nanjing 211166 P. R. China; b Shenzhen International Travel Health Care Center, Shenzhen Academy of Inspection and Quarantine Shenzhen Customs District Shenzhen 518033 P. R. China hejianan6398@163.com; c Department of Biomedical Engineering, The Chinese University of Hong Kong Hong Kong SAR P. R. China jackyfcloo@link.cuhk.edu.hk; d Shenzhen Bao'an Traditional Chinese Medicine Hospital (Group), Guangzhou University of Chinese Medicine Shenzhen 518133 P. R. China; e Department of Laboratory Medicine, Shenzhen Second People's Hospital, The First Affiliated Hospital of Shenzhen University, Health Science Center Shenzhen 518035 P. R. China wanhood@163.com

## Abstract

The coronavirus disease 2019 (COVID-19) pandemic, caused by the new severe acute respiratory syndrome coronavirus 2 (SARS-CoV-2) virus, has posed a threat to public health worldwide. Also, influenza virus has caused a large number of deaths annually. Since co-infection of SARS-CoV-2 and influenza virus, which share similar symptoms, hampers current treatment efficiency, multiple simultaneous detection of these viruses is needed to provide the right treatment for patients. We developed a microfluidic disc-direct RT-qPCR (dirRT-qPCR) assay for rapid multiplex detection of SARS-CoV-2, influenza A and B viral infection in pharyngeal swab samples in an automated manner. Choices of the DNA polymerase, concentrations of dTPs and MgCl_2_ were characterized to optimize the assay. A detection limit of 2 × 10^1^ copies per reaction was found in all three viral RNAs with as little as 2 μL of swab samples. The accuracy of our assay was evaluated with 2127 clinical swab samples of infection with these three viruses and healthy controls, and it possessed a consistency rate of 100, 99.54 and 99.25% in SARS-CoV-2, influenza A and B detection in comparison to standard RT-qPCR. The reported scheme of our assay is capable of screening other viral infections for up to 16 targets simultaneously. The whole diagnosis could be completed in 1.5 hours after simple sample loading by a non-technical expert. This constitutes an enabling strategy for large-scale point-of-care screening of multiple viral infections, which ultimately lead to a pathway for resolving the critical issue of early diagnosis for the prevention and control of viral outbreaks.

## Abstract 2

Development of a microfluidic disc-direct reverse-transcription quantitative PCR platform to perform automated multiplex nucleic acid tests for rapid multiplex detection of disease infection.

## Graphical abstract

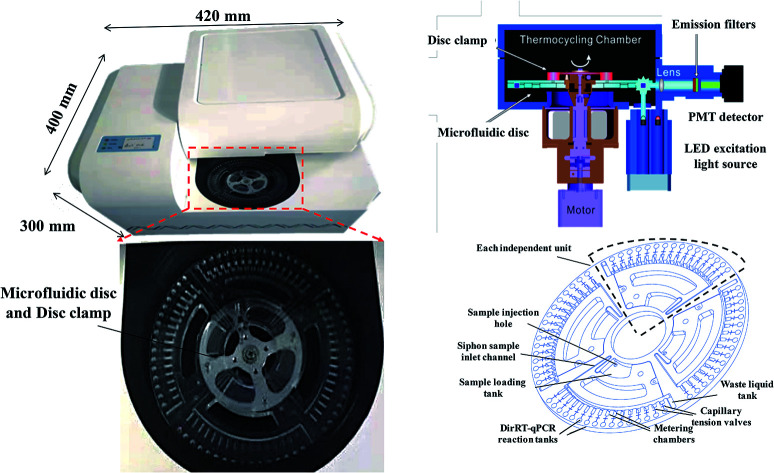

## Introduction

The coronavirus disease 2019 (COVID-19) pandemic, caused by the novel severe acute respiratory syndrome coronavirus 2 (SARS-CoV-2) virus, has posed a threat to public health worldwide in 2020, and has already resulted in more than 700 000 deaths by July 2020.^1^ Apart from this, global circulation of influenza virus type A, such as H1N1, H3N2, and type B, with symptoms very similar to COVID-19, are the primary causative agents of human acute respiratory disease and several pandemics in the last century.^2–4^ The annual influenza peak causes around 670 000 deaths worldwide. The World Health Organization (WHO) stressed the urgent need for tools, such as affordable point-of-care testing, to detect influenza for better prevention and control in a country-level approach and to serve in pandemic preparedness.^5^ These casualties can be prevented or minimized by early diagnosis prior to treatment, such as administration of anti-viral drugs, instead of relying on human immunity and passive monitoring. Yet, co-infection of SARS-CoV-2 and influenza virus hampers treatment efficiency in the worst-case scenario. Therefore, reliable multiple detection of both SARS-CoV-2 and influenza virus is of great significance in timely diagnosis to provide correct treatment of patients.^6,7^

Nucleic acid amplification tests (NAAT) are the primary method for sensitive detection of SARS-CoV-2 and influenza viral RNA. However, this detection involves multiple steps, from swab sampling to nucleic acid extraction and amplification, which are both time-consuming and labour-intensive even performed by well-trained technical experts.^8–12^ Cross-contamination and environmental pollution due to human errors could lead to false-positive or negative results in clinical samples and create unnecessary public panic.^13^ Also, the nucleic acid extraction is inefficient in samples with low viral titer.^14^ Direct polymerase chain reaction (PCR) was proposed as a rapidly emerging nucleic acid amplification detection.^15,16^ Simple sample pretreatment, instead of complicated nucleic acid extraction, such as freezing–thawing of samples, treating samples with formamide and using lysates, amplified the DNA directly from the clinical samples, *e.g.* blood. Although nucleic acid extraction and purification are skipped, these methods are still time-consuming and labourious.^17–19^ Therefore, recent optimization of sample treatment and nucleic acid amplification in the presence of inhibitors inside crude clinical samples has supported rapid viral detection.^20^ It also prevents clinical sample loss, which is critical for detecting samples of low viral titer, in each step.

However, manual operation is still required for sample mixing and reagent loading. Automatic qPCR test as a near-point-of-care or near-patient laboratory test has been described as a less time-consuming method for COVID-19 test, but such test machines are usually stationed in centralized laboratories due to its bulkiness. Inappropriate specimen shipping to laboratories is another cause of false-negative in COVID-19 test.^21,22^ Microfluidic platform (MP), also called the microfluidic chip laboratory or lab-on-a-chip (LoC), is a bioassay approach that performs fluidic manipulation in a micrometer scale. It has become a promising method for an automatic qPCR test because it miniaturizes conventional equipment for fluid actuation. Moreover, a micron-scale structure and a reaction chamber in the microfluidic chip, meaning a large surface area to volume ratio, enable faster heat transfer to shorten the thermocycling time in the RT-qPCR assays. Compared to conventional experimental techniques, the microfluidic approach offers better prospects for incorporation of integrated electronic microcontrollers, faster reaction speed and smaller sample consumption. Meanwhile, the centrifugal microfluidic platform employs a centrifugal force, instead of an external pumping system, for easy control of multiple and simultaneous fluid actuation. Also, spinning action could actuate fluid to multiple chambers under the same centrifugal force to support a highly accurate sample aliquoting. Consequently, the MP approach incurs less pollution and lower cost, making it more suitable for the high throughput and multiplex detection of pathogenic microorganisms.^23^ Various functions of conventional chemical or biological experiments have been demonstrated with the microfluidic network formed by microchannels in an automated manner.^24^ Centrifugal microfluidics have been used on clinical samples as a sample-to-answer or one-step operation strategy for detecting pathogens based on NAAT on the disc.^25–28^ Precise temperature control is critical for an effective nucleic acid amplification and it could be achieved in centrifugal microfluidics during the spinning mode.^29^ In contact-heating method, the centrifugal microfluidic disc is attached to the heating source for heat conduction, and it is desirable for isothermal DNA amplification than PCR as thermocycling speed is slow.^25^ Double-shaft turntable disc for bidirectional flow of fluid supports the actuation of the PCR reaction mixture to various temperature zones repeatedly for rapid thermocycling, and multiple of PCR discs can be placed in the main turntable disc to increase throughput.^30^ Non-contact sensing and heating for temperature regulation has simplified the embedment of the microfluidic disc onto spinning platform to support robust thermocycling without the need of complicated fluidic actuation or heating–cooling electronic components.^31^

This study primarily aims to provide an immediate solution to tackle both the current pandemic and a possible outbreak of influenza in near future. To ensure a high degree of automation and lower risk of cross-contamination, we have designed and fabricated a microfluidic platform and established the direct reverse-transcription quantitative PCR (dirRT-qPCR) assay on the microfluidic platform for multiplex detection of SARS-CoV-2, influenza A and B viruses in pharyngeal swab samples. Our optimized dirRT-qPCR assay, integrated with centrifugal microfluidics, provided a reliable direct sample detection. Compared to the conventional RNA-extraction based RT-qPCR method, the microfluidics disc-dirRT-qPCR assay has achieved high sensitivity and automation to detect SARS-CoV-2, influenza A and B viral RNA simultaneously in swab samples. Accuracy on direct detection in clinical swab samples has also been validated.

## Materials and methods

### Collection and processing of clinical samples

Clinical specimens, including 29 SARS-CoV-2, 169 influenza A and 356 influenza B positive samples, and 1572 negative samples from healthy individuals or infected with other respiratory diseases, were collected from August 2019 to May 2020 in the health and quarantine laboratory of Shenzhen International Travel Health Care Center according to the standard clinical sample collection protocol, with ethical approval and written consent from the patients and the volunteers. The samples were additionally treated with Minimal Essential Medium Eagles and Earle's Balanced Salts (MEM/EBSS) liquid broth and stored at −80 °C prior to the experiment. Standard RNA of SARS-CoV-2, influenza A and B were extracted from patient samples with the conventional RNA extraction kit as described previously.^20^

### Design of the primer pair and probes

The primer pair and the probe were synthesized by UNIMEDICA. The design of the primer pair and the probe was based on the alignment of N gene of SARS-CoV-2 (Gene ID: 43740575), conserved matrix (M1) gene of influenza A virus (Gene ID: 956527), and variant hemagglutinin (NP) gene of influenza B virus (Gene ID: 26824002) published in GenBank. The primers and the probe were designed with Primer Premier 5.0 and Primer Express 3.0.1 to generate an amplicon size of 99, 106 and 84 bp of SARS-CoV-2, influenza A and B virus respectively. The sequence of the primer pair and the hydrolysis probe, along with the sizes of the expected amplicons, were listed in Table 1.

**Table 1:** Sequences of primer pairs and hydrolysis probes used in this study

Virus	Name	Sequences (5′ → 3′)	Length (bp)
SARS-CoV-2	FP	GGGGAACTTCTCCTGCTAGAAT	22
	RP	CAGACATTTTGCTCTCAAGCTG	22
	P	FAM-TGCTGCTGCTTGACAGATT-TAMRA	19
Influenza A	FP	GACCRATCCTGTCACCTCTGAC	22
	RP	AGGGCATTYTGGACAAAKCGTCTA	24
	P	FAM-TGCAGTCCTCGCTCACTGGGCACG-BHQ1	24
Influenza B	FP	ATTCCAATTAAGCARACCATCC	22
	RP	GTTGCTTTAATAATCGAGGTCATC	24
	P	VIC-TTGGGAGGGACACAGCAGAGGAT-BHQ1	23

### Centrifugal microfluidic platform

**Fig. 1:**
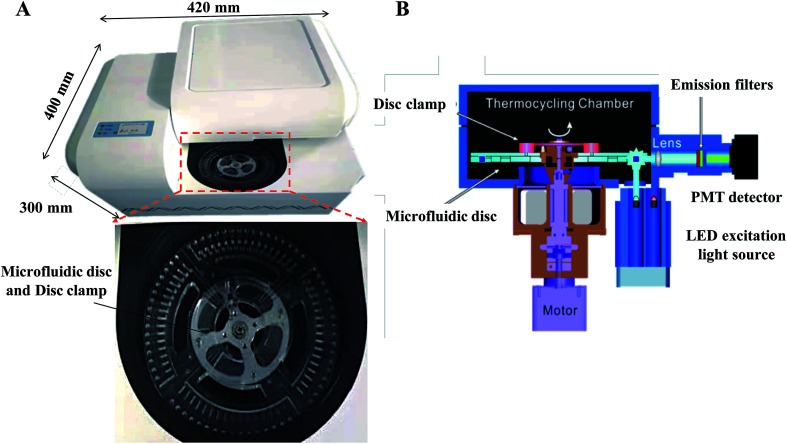
(A) Image of the constructed centrifugal microfluidic equipment and magnified view of the microfluidic cassette. The diameter of the disk is 125 mm, and the reaction tank is located on the periphery. (B) Schematic diagram showing the spinning, thermocycling and optical detection in the microfluidic equipment.

Our centrifugal microfluidic platform consists of the following compartments: a microfluidic disc layer, an optical detection unit, a temperature control unit, and a mechanical control unit for both nucleic acid amplification and real-time fluorescence detection. Fig. 1A shows the image of the machine layout and a magnified view of the microfluidic layer. Fig. 1B is the schematic diagram showing the light path in an optical detection unit, regulated heat flow in the temperature control unit, and a motorized stage for disc spinning in the mechanical control unit. Air flow for heating and cooling was employed to allow a rapid temperature thermocycling to shorten the time of RT-qPCR. Incident LED light excitation of 494 and 538 nm wavelength and a photomultiplier tube (PMT) light detector with a bandpass filter for 518 and 554 nm were used for collection of the fluorescence emission light in this experiment.

**Fig. 2:**
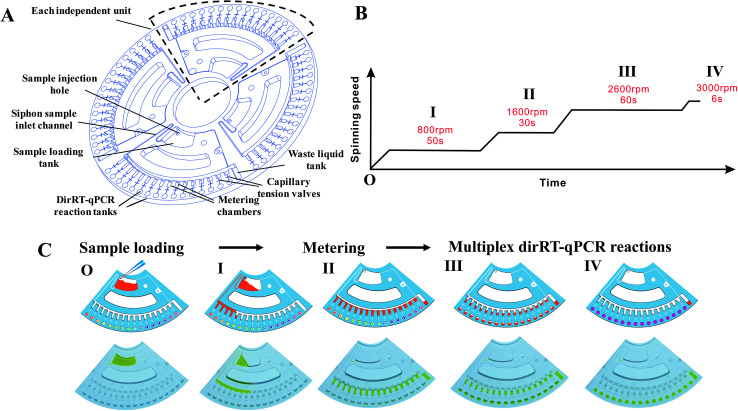
Microfluidic disc configuration and spinning profile in the microfluidic platform. (A) Schematics of the microfluidic disc showing four independent units in a single disc, with all of the features and the chambers labelled. (B) Configuration and (C) characterization of the spinning profile of the designed disc in our microfluidic platform. Schematic diagram (upper) and photos (lower) show the sequential step of the fluid actuation under spinning program.

For the fabrication of microfluidic disc, injection molding technology with a molding angle of 3° on transparent PMMA was used to mass-produce the base microfluidic disc layer, which provides the microfluidic network and the reaction vessel for the fluid actuation. Fig. 2A shows the design of the disc with a diameter of 120 mm and thickness of 3 mm. The microfluidic system is divided into four independent reaction units and each unit accounts for a quarter of the disk area. Each unit includes a sample-loading hole and a sample-loading tank for mixing of sample and dirRT-qPCR reaction mixture, a siphon sample inlet channel, metering chambers, a series of capillary tension valves, PCR reaction tanks, a waste liquid tank. The designed microfluidic disc in our work can simultaneously detect four clinical samples, and 16 targets of each sample can be evaluated at the same time. After pre-loading of primer pairs, probes and dirRT-qPCR reaction mixture, the base layer is sealed with a transparent film on top, for the ease of observation of fluidic flow, and stored at 4 °C until use.

### Microfluidic disc-dirRT-qPCR assay

Four DNA polymerases, PrimeDirect (Takara), OmniTaq (DNA Polymerase Technology Inc), Alpha Taq (VitaNavi) and TTX (TOYOBO), which are mutants of DNA polymerase resistant to the inhibitory effect of blood, were selected to evaluate the efficiency of our microfluidic disc-dirRT-qPCR assay. The initial PCR conditions were established according to the melting temperature (*T*_m_) value of the primer pairs and the probe and the recommended reaction temperature of the DNA polymerases. We selected the reaction mixture containing DNA polymerase, a primer pair, a probe, and a sample template with optimized concentrations of MgCl_2_ and dNTPs. The microfluidic disc-dirRT-qPCR reactions were performed in our microfluidic machine with a volume of 20 μL each and capable for, at maximum, 16 simultaneous target detection. Each experiment was repeated in triplicate. Positive control and no template control (NTC) were included in each experiment. PrimeDirect polymerase was selected for the downstream PCR assay with the corresponding reaction procedure shown as follows: reverse transcription was performed at 90 °C for 3 min and 60 °C for 5 min for RT reaction, then 95 °C for 5 s, followed by 60 °C for 30 s, and repeated for 40 cycles. To validate the accuracy of the nucleic acid amplification, agarose gel electrophoresis was performed on the samples after 30 and 40 cycles of reaction. The gels were imaged to evaluate the amplicon size and number of the bands. To validate the practical performance, sensitivity and accuracy of this assay were evaluated using the standard viral samples and clinical swab samples.

### Statistical analysis

Statistical analyses were conducted using OriginPro (version 2018) and GraphPad Prism (version 5). The sensitivity and reliability of the dirRT-qPCR assay were determined by analyzing the mean cycle threshold (Ct) values and standard deviations (SD) of Ct values with that in conventional RT-qPCR assay.

## Results

### Validation of the sample-to-answer multiplex assays on the centrifugal microfluidic platform

Fluidic actuation inside the disc was first characterized before integrating the dirRT-qPCR assay into it for thermocycling reaction and fluorescence detection. We adjusted the rotating speed based on the design of fluidic channels on the microfluidic disc to ensure the fluidic flow is highly consistent. Microfluidic-disc dirRT-qPCR experiment was performed on the disc with the automated spinning profile in Fig. 2B. The schematic diagram (upper) and photos (lower) of each fluidic actuation step in our centrifugal microfluidic are shown in Fig. 2C. The unpinned disc was first increased to 800 rpm for 50 s to draw the loaded samples, mixed with dirRT-PCR reaction cocktails, from the sample-loading tank to the metering chamber through a siphon valve. It was increased to 1600 rpm for 30 s to pass all the excess samples after metering to the waste chamber. It was then increased to 2600 rpm for 60 s to overcome the resistance of the capillary tension valve and flow to the dirRT-qPCR reaction tanks, pre-loaded with primer pairs and probes, for the target multiplex viral detection. It was increased to 3000 rpm for 6 s to ensure all the metered fluid flowed into the reaction tanks. The centrifugal speed was set as 400 rpm to hold the fluids inside the reaction tanks for the subsequent dirRT-qPCR reactions. The total centrifugation time for sample mixing, metering and loading to multiplex reaction sites was within 180 s.

### Sensitivity of the microfluidic-disc multiplex dirRT-qPCR assay

**Fig. 3:**
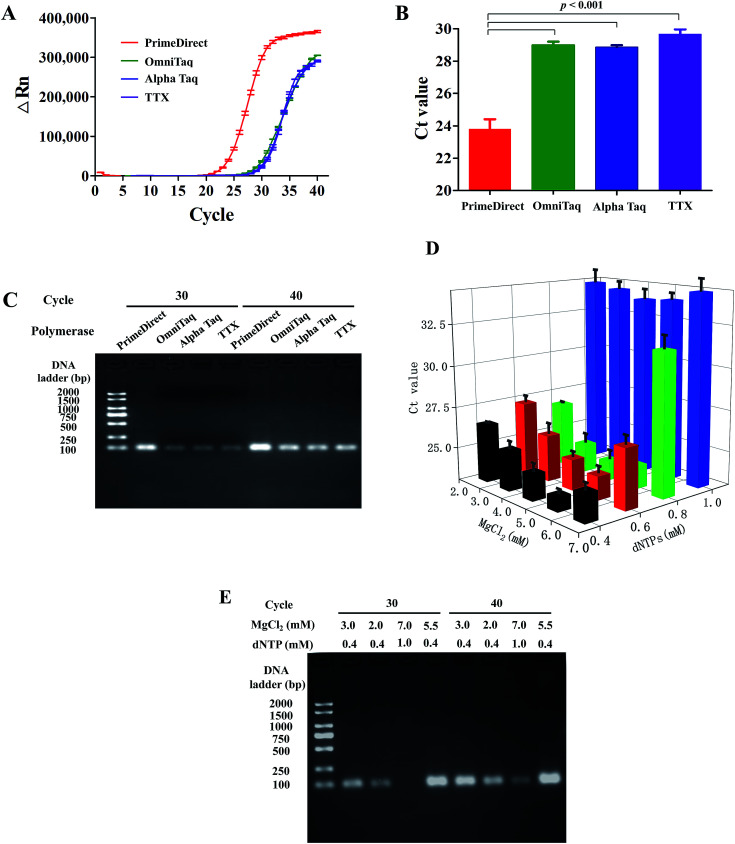
Optimization of the reaction mixture in microfluidic disc-dirRT-PCR to achieve effective detection in the swab samples. (A) Amplification curves and (B) statistical Ct analysis shows the amplification efficiency of four selected DNA polymerases, *i.e.* PrimeDirect, OmniTaq, Alpha Taq, and TTX on the standard viral RNAs-sparked swab samples. (C) Image of gel electrophoresis of end-point dirRT-PCR using four polymerases at both cycle 30 and 40. (D) Summary of Ct analysis using PrimeDirect polymerase and varying MgCl_2_ (2.5–6.5 mM) and dNTPs (0.4–1.0 mM) concentrations in the reaction mixture. (E) Image of gel electrophoresis of end-point dirRT-PCR followed with gel electrophoresis on four selected conditions (unoptimized concentrations of dNTPs and MgCl_2_, lowest concentrations of dNTPs and MgCl_2_, highest concentrations of dNTPs and MgCl_2_, optimized concentrations of dNTPs and MgCl_2_) at both cycle 30 and 40.

After characterization of fluid actuation in our centrifugal microfluidic disc, dirRT-qPCR was integrated into the reaction on our microfluidic platform and its performance was evaluated. Four commercially available polymerases (PrimeDirect, OmniTaq, Alpha Taq, and TTX) that claimed to work on clinical samples, such as blood, for direct PCR were included in our assay. Amplification efficiency was evaluated by both the amplification curves and the Ct value of these four reaction mixtures with the standard viral RNAs-sparked swab samples (Fig. 3). All of them worked in our dirRT-qPCR assay, showing positive signals with a mean Ct below 30. Also, amplification efficiency of PrimeDirect polymerase was the highest, with its lower Ct value and higher overall fluorescence signals with time, out of the four polymerases (Fig. 3A). Further statistical analysis (Fig. 3B) shows that the use of PrimeDirect polymerase outweighed the other polymerases and provided faster detection. Amplification efficiency was further characterized using end-point dirRT-PCR followed with gel electrophoresis, which could provide not only evidence on amplification efficiency, determined by the intensity of the bands, but also its accuracy on amplifying the correct amplicon size. Fig. 3C shows the gel electrophoresis of the dirRT-qPCR assay with four polymerases at both cycle 30 and 40. All polymerases could amplify the correct amplicon size without amplifying other regions or potential primer-dimer regions. PrimeDirect polymerase showed a brighter band compared to other polymerases at both cycle 30 and 40. Therefore, PrimeDirect polymerase was selected for the downstream experiments.

Apart from the evaluation of the polymerases to select the best one for our dirRT-qPCR assay, we have characterized the dirRT-qPCR reagent used, including the essential cofactor magnesium ions (Mg^2+^) and substrate dNTP for polymerase to construct the amplicon, on both amplification efficiency and accuracy. In addition, the optimized concentration of these components is critical to reducing the false negative by dNTPs, with a chelating effect to interact with magnesium ions, at non-optimal high concentrations to inhibit dirRT-qPCR reaction. To determine the optimal concentrations of dNTPs and MgCl_2_, the dirRT-qPCR was performed with PrimeDirect polymerase and the reaction mixture and a combination of various concentrations of MgCl_2_ (2.5–6.5 mM) and dNTPs (0.4–1.0 mM). To evaluate the amplification efficiency, the Ct values were summarized in three-dimensional bar graphs in Fig. 3D. The optimal concentrations of dNTPs and MgCl_2_ were determined by the lowest Ct value, indicating the highest amplification efficiency. The increase in the concentration of MgCl_2_ showed an insignificant improvement in amplification efficiency, while a high concentration of dNTPs inhibited the amplification at a low concentration of MgCl_2_. The optimal concentration of dNTPs and MgCl_2_ were 0.4 and 5.5 mM, respectively in our system. Fig. 3D showed the Ct values showing the optimization of the concentration of dNTPs and MgCl_2_ used in assay on the pharyngeal swabs. End-point dirRT-PCR followed with gel electrophoresis was performed on four selected conditions (un-optimized concentrations of dNTPs and MgCl_2_, lowest concentrations of dNTPs and MgCl_2_, highest concentrations of dNTPs and MgCl_2_, optimized concentrations of dNTPs and MgCl_2_) to further validate the result in Fig. 3D. Fig. 3E showed clearly brighter bands of correct amplicon size on the dirRT-PCR with optimized concentrations of dNTPs and MgCl_2_ at both cycle 30 and 40.

**Fig. 4:**
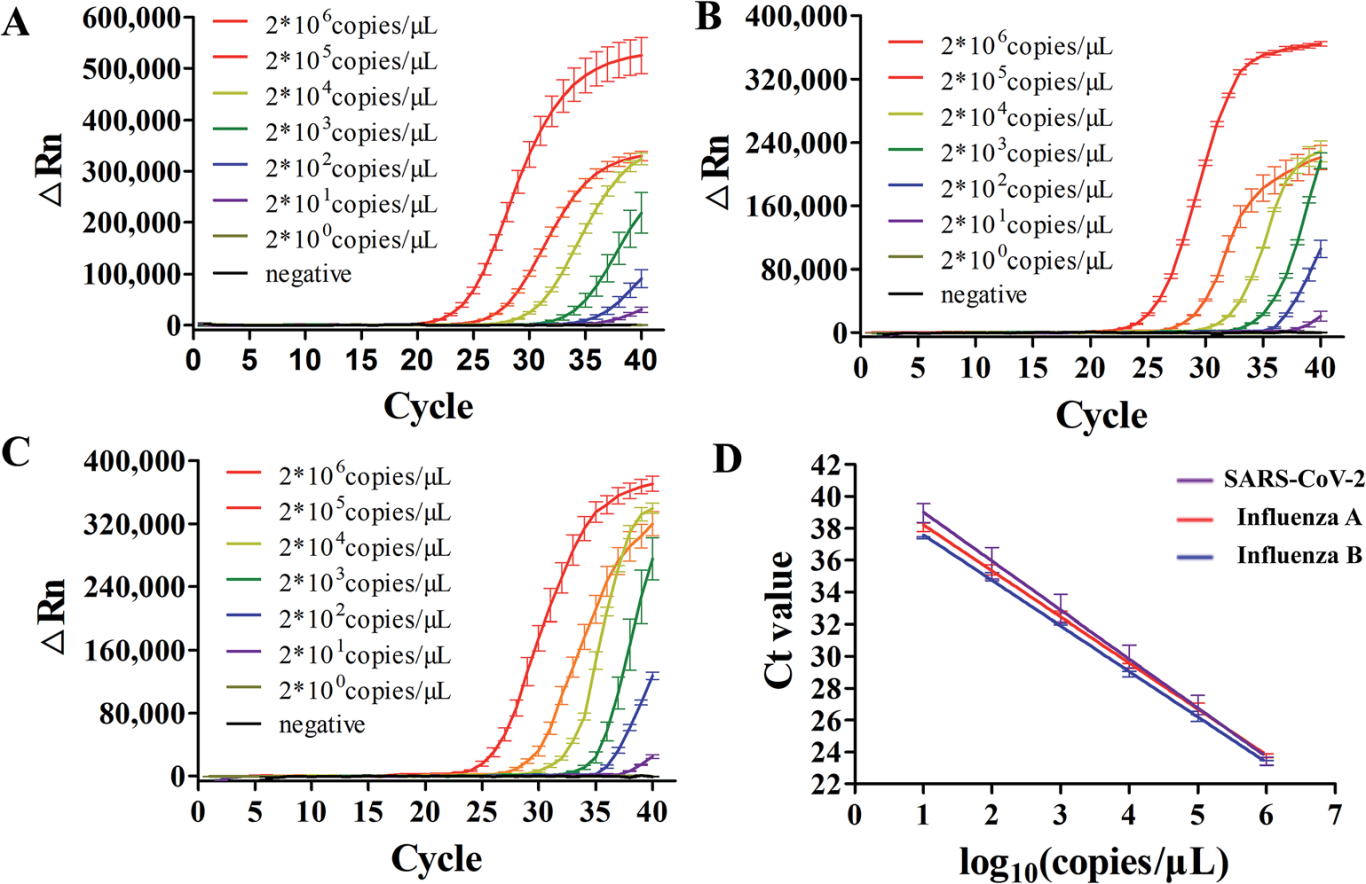
The sensitivity of the microfluidic disc-dirRT-qPCR assay in multiplex detection of SARS-CoV-2, influenza A and B. (A–C) Amplification curves using standard viral RNAs of (A) SARS-CoV-2, (B) influenza A and (C) influenza B sparked swab samples. (D) Standard curve of mean Ct with standard viral RNAs concentrations of the three viruses.

After the optimal condition was established, we evaluated its sensitivity in detecting standard concentrations of three viral RNAs sparked in swab samples. It was first evaluated using simulated clinical samples with standard viral RNAs ranging from 2 × 10^0^ to 2 × 10^6^ copies per reaction. There were amplification curves in all the concentrations when compared to negative (black curve) in SARS-CoV-2, influenza A and B screening (Fig. 4A–C), suggesting that this dirRT-qPCR on the microfluidic platform could be performed with direct sample addition without sample pretreatment. The high linearity (*R*^2^ = 0.99) of all three standard curves (Fig. 4D) supports the use of dirRT-qPCR assay for quantification of viral RNA copies number ranging from 2 × 10^1^ to 2 × 10^6^ copies per reaction in clinical infection samples. The detection limits were 2 × 10^1^ copies per reaction in all three viruses (Table 2).

**Table 2:** Summary of sensitivity of microfluidic-disc dirRT-qPCR assay

Virus	Dynamic range	Standard curve	Mean Ct ± SD	Detection limit (copies per reaction)
SARS-CoV-2	2 × 10^1^ to 2 × 10^6^	*y* = −3.27*x* + 43.50	23.43 ± 0.24	2 × 10^1^
Influenza A	2 × 10^1^ to 2 × 10^6^	*y* = −2.90*x* + 41.137	23.68 ± 0.11	2 × 10^1^
Influenza B	2 × 10^1^ to 2 × 10^6^	*y* = −2.87*x* + 40.495	23.18 ± 0.15	2 × 10^1^

### Clinical evaluation of the microfluidic disc-dirRT-qPCR assay on clinical samples

**Fig. 5:**
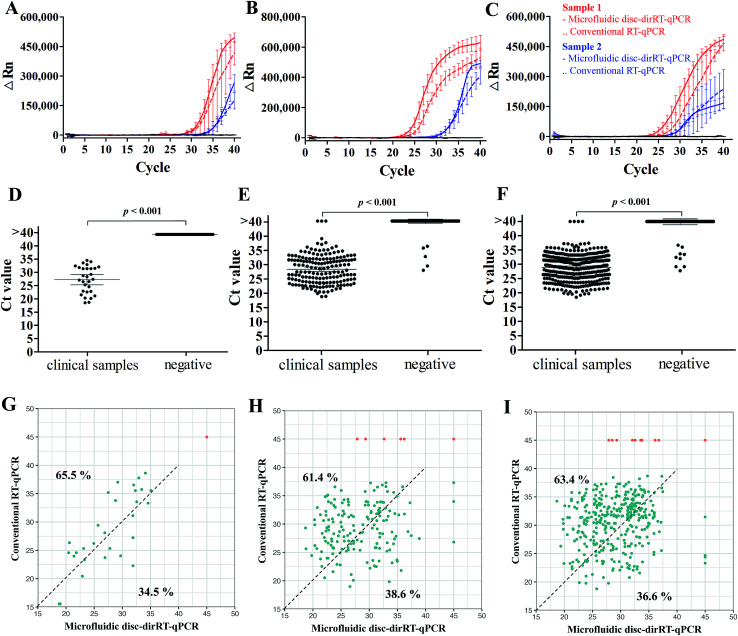
Accuracy of clinical detection with our microfluidic dirRT-qPCR compared to standard RNA-extraction based RT-qPCR for multiplex detection of SARS-CoV-2, influenza A and B viruses in clinical infectious swab samples. Typical amplification curves of our microfluidic-disc-dirRT-qPCR (solid lines) and standard RNA-extraction based RT-qPCR (dash lines) from two patients (red and blue) and Ct analysis on effective screening of patients infected with (A and D) SARS-CoV-2, (B and E) influenza A, (C and F) influenza B as well as healthy individuals as the negative control. (G–I) Relationship of Ct between our microfluidic-disc-dirRT-qPCR (*x*-axis) and conventional RT-qPCR (*y*-axis) of each positive (blue) or negative (orange) sample for (G) SARS-CoV-2, (H) influenza A and (I) influenza B screening. Percentages of the positive sample (blue dots) shifted in upper left and lower right regions were reported.

After understanding its sensitivity and dynamic range of our microfluidic disc-dirRT-qPCR assay, we employed it for screening samples with clinical infection of these three viruses. The accuracy was evaluated by comparing our established microfluidic disc-dirRT-qPCR assay and the current standard RNA-extraction based RT-qPCR on clinical specimens of these viral infections. Fig. 5A–C shows typical amplification curves of our established microfluidic-disc-dirRT-qPCR (solid lines) and standard RNA-extraction based RT-qPCR (dash lines) from two patients (red and blue lines) infected with the indicated disease. The overlapping curves of solid and dash lines of each patient suggest the amplification rate is similar between our microfluidic disc-dirRT-qPCR and conventional RT-qPCR and thus resulted in similar Ct values for disease screening. Fig. 5D–F show a statistically significant difference in the Ct value between 29 positive samples of the three types of virus infection compared to the negative controls. It therefore supports the use of our platform as a potential standard method for effective screening of positive/negative disease infection. 40 cycles with sample pre-processing in our microfluidic platform only requires as short as 90 min. The mean Ct value were 27.71, 28.20 and 29.80 in SARS-CoV-2, influenza A and B respectively. It suggested that positive results can be confirmed in 57 min after sample loading in our assay.

Furthermore, the evaluation of the accuracy, an important criterion for effective biosensing, has been done with 2127 clinical swab samples of infection with the three viruses and healthy controls in comparison to the current gold standard, *i.e.* conventional RNA-extraction based RT-qPCR detection to support the reliability of our methods in practical use. Table 3 shows the summary of larger-scale sample analysis in screening patients infected with SARS-CoV-2, influenza A and B with a total of 2127 clinical samples (29 SARS-CoV-2-positive samples, 171 influence A-positive samples, 355 influence B-positive samples), and 1572 negative samples from healthy individuals or infected with other respiratory diseases were used in a larger-scale sample analysis, and it showed 100%, 99.54% and 99.25% accuracy on the diagnosis of SARS-CoV-2, influenza A and B respectively. Positive predictive value (PPV) of detecting SARS-CoV-2, influenza A and B are 100%, 97.11% and 97.75% and negative predictive value (NPV) of detecting them are 100%, 99.81%, and 99.71% respectively. To show the consistency of these methods on clinical detection, Fig. 5G–I shows the relationship of Ct between our microfluidic-disc-dirRT-qPCR (*x*-axis) and conventional RT-qPCR (*y*-axis) in each positive (blue) or negative (orange) sample for SARS-CoV-2, influenza A and B screening, respectively. The positive sample (blue dots) tends to shift in upper left region (65.5%, 61.4% and 63.4% for SARS-CoV-2, influenza A and B screening respectively) indicated smaller Ct values were found in our microfluidic-disc-dirRT-qPCR. Therefore, our nucleic amplification assay of this paper is a less time-consuming method compared to standard RNA-extraction based RT-qPCR. Apart from that, Table 4 showed the summary of operational processes with our microfluidic disc-dirRT-qPCR compared to standard RNA-extraction based RT-qPCR.^32–36^ The overall reduction in time and reagent loading and downstream processes support the use of our method over standard RNA-extraction based RT-qPCR.

**Table 3:** Summary of evaluation on the accuracy of clinical detection with our microfluidic disc-dirRT-qPCR compared to standard RNA-extraction based RT-qPCR

Type of viral infection	Microfluidic disc-dirRT-qPCR	Conventional RT-qPCR		Total
		Positive	Negative	
SARS-CoV-2	Positive	29	0	29
	Negative	0	74	74
	Total	29	74	103
Influenza A	Positive	168	5	173
	Negative	3	1567	1570
	Total	171	1572	1743
Influenza B	Positive	351	9	360
	Negative	4	1379	1383
	Total	355	1388	1743

**Table 4:** Summary of operational processes with our microfluidic disc-dirRT-qPCR compared to standard RNA-extraction based RT-qPCR

Methods	Microfluidic disc-dirRT-qPCR	Conventional RT-qPCR
Reagent loading	10 min	30–60 min^32–34^
RNA extraction	—	30–120 min^32–34^
Reverse transcription	—	30 min^35^
Nucleic acid amplification	80 min (combined with reverse transcription)	60–90 min^32–34,36^

## Discussion

The novelty of this work lies in the implementation of our automated centrifugal microfluidic platform and optimized dirRT-qPCR reaction for sample-to-answer diagnosis of multiple viral infections from unpurified clinical pharyngeal swab sample. It offers a simple operating process, *i.e.* loading swab samples onto the disc, as well as high sensitivity and accuracy to demonstrate a simultaneous screening of clinical SARS-CoV-2, influenza A and B virus infection. The entire detection process can be completed within 1.5 hours, and positive signals can be detected in 57 min, making it at least three times faster with the conventional RT-qPCR approach.^37^ The test can be conducted with a very small sample volume, thus greatly reducing the risk of human contact during sample collection. Moreover, the use of small sample volume also facilitates its application in situations where the availability of sample is relatively scarce. The reported centrifugal microfluidic platforms could provide the simple processes in an automated manner, similar to other reported centrifugal microfluidic systems, but our integration with dirRT-qPCR assay with conditions optimized could further simplify the design of microfluidic disc and reduce the processes to be executed during disc spinning, thus increasing the robustness and accuracy for clinical use.

In recent years, microfluidic-based assays and direct PCR amplification have gained popularity, since they reduce laborious procedures and time needed for the extraction and purification of nucleic acid and sample aliquoting for multiple detection. The main problem in direct PCR amplification technique, where nucleic acid extraction and purification are omitted, is the inhibition effect by PCR inhibitors commonly found in human specimens, such as hemoglobin and lactoferrin in blood, immunoglobulin G (IgG) present in plasma, urea in urine and complex polysaccharides in faeces.^38–40^ This reported work is mainly focused on swab as it has been considered as a better minimally invasive method than blood for sampling. Pharyngeal swabs, the most common sample acquired, are believed to contain fewer PCR inhibitors and are relatively simple to collect than blood or plasma. Non-invasive saliva collection is also considered as an alternative to replacing invasive venipuncture for virus diagnosis, as a recent study showed that saliva is a more reliable tool for the detection of SARS-CoV-2.^41^ It was also observed that positive detection of influenza viral genetic markers in the pharyngeal swab samples was only possible within two days after the onset of the disease, while detection in saliva was possible for at least four days on average. The range of RNA viral load in saliva is 10^4.1^ to 10^7.4^ copies per mL.^42^ Therefore, NAAT has shown both good sensitivity and specificity for influenza screening, where nearly both 100% PPV and NPV could be achieved using different combinations of primers, probes and RT-qPCR cocktails.^43,44^ Although our primers targeting SARS-CoV-2 viral sequence was established recently, a higher specificity lies in a more comprehensive genome analysis of the virus. Unfortunately, the high mutation rate of RNA-based virus is one of the reasons of false negative in NAAT, where the primer or the probe-binding site does not match the mutated viral sequence. Addition of COVID-19-RdRp/Hel was evaluated lately to provide a higher specificity.^45^ It can be incorporated into our multiplex detection system without affecting the workflow. In our platform, the high dynamic range, from 2 × 10^1^ to 2 × 10^6^ copies per reaction, supports diagnosis of viral infection in different stages. Viral load in patients with SARS-CoV-2 infection ranged from 10^2^ to 10^6^ copies per sample in different phases of infection and sample types. High viral load was found in samples of asymptomatic patients.^46^ Therefore, our platform could provide not only screening in suspected cases for early diagnosis, but large-scale screening to find disease carriers. It is worth noting that viral shedding may start before the onset of symptoms and therefore creates difficulties in accurate detection. Complete SARS-CoV-2 viral shedding may occur after a median of 20 days, with Ct > 40 that leads to false negative in RT-qPCR assay.^47^

The detection time of other methods that omit RNA extraction can be ranging from 0.5–3.5 hours, for example, the SHERLOCK method takes 2 hours,^48^ while the heat treatment approach takes 3.5 hours.^49^ Other nucleic acid amplification strategies, such as isothermal DNA amplification, instead of PCR, can also be used to further increase its sensitivity, specificity and shorten its reaction time.^50^ Although LAMP-based isothermal amplification on SARS-CoV-2 can be completed within 0.5 hours, it is not suitable for quantification, which is important to evaluate the severity of the infection and potential transmission rate of the patient for disease control.^51^ Moreover, our platform can be translated to detecting other biomarkers rather than viral RNA. One example is human microRNAs, which are upregulated or downregulated in different disease infections, as alternative biomarkers to tackle the problem of false-negative in targeting RNA virus with high mutation issue.^52,53^ Apart from detecting nucleic acid for disease diagnosis, our centrifugal microfluidic platform could be used in protein-based immunoassays to provide additional information of infection status by screening viral surface protein antigens, or seroconversion, *i.e.* the extent and duration of immunity to the virus, by screening human antibodies regenerated after infection, where the latter is particularly useful for longitudinal serological studies in post-pandemic monitoring.^54^ The multiplex immunoassay based on multiple lateral flow paper strip assays has been demonstrated to extend its rapid, automated and simultaneous detection of multiple kinds of pathogen surface antigen with the use of centrifugal microfluidics.^55,56^

With the ongoing COVID-19 pandemic, the WHO expected that the COVID-19 may become an endemic disease and constant outbreaks may be common in the future. At the same time, the uncontrolled spread of influenza A and B viruses in high-population areas has been an emerging issue. Influenza season in the fall–winter period contributes to a significant amount of disease-caused death, and as its symptoms are similar to COVID-19 it may create public panic. In addition, since lockdown measures of different countries will be loosened in the coming months, reliable continuous monitoring of the virus is needed among cross-border individuals, especially children, who had a high incidence of other common coronavirus infections and were more likely to be infected and transmit viruses across the border, and the elderly, who in general have a weaker immune system.^57^ Our microfluidic disc-dirRT-qPCR with its platform could achieve a more straightforward and convenient detection as an alternative to the current standard RNA-based RT-qPCR method. Our platform enables the high-throughput sample-to-answer multiplex detection of SARS-CoV-2, influenza A and B viruses and other emerging infectious diseases, supporting point-of-care diagnosis for on-site screening. It ultimately realizes the goal of large-scale screening of viral infection for early diagnosis and the prevention and control of viral outbreaks for post-pandemic prevention and monitoring.

## Conflicts of interest

The authors declare no conflict of interest.

## Supplementary Material
